# Tunable TiZrMoC Coatings: A Comprehensive Study of Microstructure, Mechanical Properties, and Wear Resistance

**DOI:** 10.3390/nano14241986

**Published:** 2024-12-11

**Authors:** Alexander Pogrebnjak, Volodymyr Buranych, Volodymyr Ivashchenko, Svitlana Borba-Pogrebnjak, Olga Maksakova, Maria Caplovicová, Alexander Goncharov, Alexei Onoprienko, Petro Skrynskyy, Martin Sahul, Piotr Konarski, Piotr Budzynski, Mariusz Kaminski, Marek Opielak, Dominik Flock, Vasiliy Pelenovich, Yang Bing

**Affiliations:** 1 Biomedical Research Centre, Sumy State University, 116, Kharkivska St., 40007 Sumy, Ukraine; borbac@i.ua (S.B.-P.); o.goncharov@mss.sumdu.edu.ua (A.G.); 2 Institute of Materials Science, Faculty of Materials Science and Technology, Slovak University of Technology, J. Bottu 25, 917 24 Trnava, Slovakia; volodymyr.buranych@stuba.sk (V.B.); maksakova.tereshenko@gmail.com (O.M.); martin.sahul@stuba.sk (M.S.); 3 Frantsevich Institute for Problems of Materials Sciences, NAS of Ukraine, Krzhyzhanovsky 3, 03142 Kyiv, Ukraine; dir@ipms.kyiv.ua (V.I.); onopr@ipms.kiev.ua (A.O.); p.scryn@ipms.kyiv.ua (P.S.); 4 Centre for Nanodiagnostics of Materials, Slovak University of Technology in Bratislava, Vazovova 5, 812 43 Bratislava, Slovakia; maria.caplovicova@stuba.sk; 5 Łukasiewicz Research Network–Tele and Radio Research Institute, 11, Ratuszowa St., 03-450 Warsaw, Poland; piotr.konarski@itr.lukasiewicz.gov.pl; 6 Faculty of Mechanical Engineering, Lublin University of Technology, Nadbystrzycka Str. 36, 20-618 Lublin, Poland; p.budzynski@pollub.pl (P.B.); mariusz.kaminski@pollub.pl (M.K.); 7 Faculty of Transportation and Information Technology, WSEI University, 4, Projektowa Str., 20-209 Lublin, Poland; m.opielak@pollub.pl; 8 Institute of Materials Science and Engineering, Ilmenau University of Technology, Gustav-Kirchhoff Str. 1, 98693 Ilmenau, Germany; dominik.flock@tu-ilmenau.de; 9 Hubei Key Laboratory of Electronic Manufacturing and Packaging Integration, Wuhan University, Wuhan 430072, China; pelenovich@mail.ru; 10 Institute of Technological Sciences, Wuhan University, Wuhan 430072, China; 11 School of Power & Mechanical Engineering, Wuhan University, Wuhan 430072, China; 00008360@whu.edu.cn

**Keywords:** TiZrMoC coatings, dual DC magnetron sputtering, X-ray photoelectron spectroscopy, computational modeling, nanostructure

## Abstract

TiZrMoC coatings were deposited on Si(100) substrates using a DC dual magnetron sputtering. The composition was controlled by adjusting the sputtering parameters of the TiZrMo and graphite targets. The influence of graphite target current on the resulting coating properties was explored. TEM analysis revealed a single-phase structure with Ti/Mo/Zr substitutional elements, columnar grains, and a strong [111] texture. Nanotwins and stacking faults were prevalent within the nanocrystals. EDX, SIMS, XRD, and XPS analyses confirmed the elemental composition and nanostructure. Computational modeling was employed to investigate the mixing behavior of the quaternary solid solutions depending on the valency electron concentration. The films exhibited exceptional mechanical properties, including a maximum hardness of 35 GPa and a wear rate of 2.11 × 10^−7^ mm^3^N^−1^m^−1^, attributed to the presence of an amorphous carbon layer and optimized deposition parameters. These findings demonstrate the potential of TiZrMoC coatings for advanced applications requiring exceptional wear resistance and durability.

## 1. Introduction

Understanding and enhancing the properties of surfaces is pivotal in advancing material applications across various industries. Titanium alloy composite coatings are highly valued for their mechanical properties, cost-effectiveness, and resistance to wear and corrosion [1,2]. Ongoing research on transition metal (TM) carbide systems, particularly TiC coatings, has uncovered their potential [3,4,5,6]. One significant aspect of transition metal carbides is their ability to form TM1-TM2-C solid solutions [7,8,9,10], improving their functional properties. Studies show a strong connection between hardness and valence electron concentration (VEC), suggesting a peak in hardness for specific carbides around VEC ≈ 8.4 [11]. Microhardness measurements [12] confirm this trend, highlighting optimized mechanical properties within a certain composition range. First-principles calculations [13] delve into the stability and mechanical attributes of ternary alloys, predicting maximum hardness for compositions with VECs around 8.5 to 8.75. The examination of surface composition, specifically the arrangement of elements and phases, provides essential insights into the connection between VEC and material characteristics in ceramic systems based on transition metals. Methods like X-ray photoelectron spectroscopy (XPS) offer insights into the electronic configuration and chemical interactions, illuminating the impact of VEC on electron density distribution surrounding metal atoms on the surface. This understanding, combined with theoretical modeling, allows for the investigation of how changes in VEC influence the durability of metal-carbon bonds, phase stability, and ultimately, the mechanical and tribological properties [14,15,16]. Combining surface observations with bulk property analyses provides a comprehensive understanding of how VEC influences the performance of transition metal-based ceramic systems at the nanoscale and macroscale.

Among the variety of TM-based materials, the alloys in the TiZrMo system (known in the literature as a TZM) have attracted considerable attention from researchers. TZM alloys play a crucial role in industries like aerospace and automotive industry, where durability and performance are top priorities [2,8,15,17,18,19,20,21,22]. For instance, in aerospace, they increase the lifespan of turbine blades, while in the automotive sector, they boost engine efficiency. Zirconium is in the same chemical group as titanium and can improve mechanical strength and long-term efficiency in binary TiZr systems [23,24,25]. Molybdenum is a strong β-stabilizer, which can reduce Young’s modulus and enhance corrosion resistance in binary TiMo systems [26,27,28]. In this context, the TZM alloys exhibit a high melting point, strength, modulus of elasticity, low vapor pressure, good thermal conductivity, corrosion resistance, and excellent high-temperature strength. These properties make TZM alloys valuable in various strategic technologies and sectors. To our knowledge, studies on the TiZrMo system have primarily focused on bulk alloys, and significant research on coatings is limited, except for the work by Marupalli et al. [21].

When exploring the surface properties of nanostructured TiZrMoC coatings, the influence of elemental doping on nucleation and growth mechanisms plays an important role. Studies on multicomponent ceramic systems [29,30,31,32,33] have shown that multicomponent carbides, such as those based on Nb, promote carbide nucleation by mitigating mismatch strains and interfacial energies, thus facilitating carbide formation. Moreover, variations in Ti content within the coatings lead to changes in carbide size and quantity, consequently affecting wear resistance [33]. Coatings with higher Ti content exhibit less wear compared to those with lower Ti content, although Ti-free coatings exhibit the least wear overall. Additionally, the introduction of Ti enhances the stability of interfaces, with distinct covalent bonds forming at the steel substrate-M interface and ionic bonds at the M-C interface. These findings underscore the critical role of elemental composition in determining the surface properties and performance of nanostructured coatings.

TiZrMoC coatings hold immense potential for several industrial applications [19,21] due to their unique combination of tribomechanical properties and stability in high-temperature and corrosive environments. In the aerospace and automotive industries, these coatings can significantly enhance the service life of engine components: turbine and compressor blades, combustion chamber parts, seal rings, cylinder liners, etc. [34,35,36]. Similarly, in the energy sector, TiZrMoC coatings are valuable for components in advanced power generation systems, such as steam turbines and heat exchangers, where stable functionality and longevity are critical [37]. Furthermore, in industrial manufacturing, TiZrMoC coatings can be applied to machining tools and molds, reducing downtime and costs in metal forming and injection molding as an example of binary TiC [38], ZrC [39], and MoC [40]. By combining the individual strengths of titanium, zirconium, molybdenum, and carbon, these coatings represent a significant advancement in material resilience and efficiency across various industries.

Given the details mentioned above, it is useful to analyze complex TZM ceramic systems. Therefore, the core objective of this study was to investigate the potential of novel material by examining the TiZrMoC coatings deposited through the dual DC magnetron sputtering technique, utilizing TiZrMo and graphite targets. In this study, we characterize physical and chemical characteristics to understand the formation of mechanical and tribological properties. We aimed to fill the gap in studying the quaternary carbide systems, at least partially. So, we have carried out first-principles investigations of the stability, elastic properties, Vickers hardness, and fracture toughness of the random cubic quaternary TM1_0.25_TM2_0.25_TM3_0.5_C and TM1_0.33_TM2_0.33_TM3_0.33_C (TM1, TM2, TM3 = Ti, Zr, Mo) solid solutions as well the ternary carbide alloys Ti_0.5_Zr_0.5_C, Ti_0.5_Mo_0.5_C, and Zr_0.5_Mo_0.5_C. The results of the calculations were used to interpret the properties of the deposited coatings.

## 2. Materials and Methods

### 2.1. Coating Deposition

The TiZrMoC coatings were applied in a deposition unit that featured two magnetrons positioned horizontally, enabling the concurrent co-sputtering of two targets. These targets were set up at an angle of 86° relative to each other. The rotating substrate holder was positioned in the plane where the fluxes from the sputtered targets intersected. This configuration of fixtures enabled the manipulation of coating composition by adjusting specific sputtering parameters and ensured an even dispersion of sputtered elements across the substrate surface. Further information about the deposition unit’s specifics is available in another article [41]. The targets utilized were disks with a diameter of 72 mm and a thickness of 4 mm. The TiZrMo target (Ti_52_Zr_32_Mo_16_, EDX data) was manufactured by vacuum arc melting in the atmosphere of high-purity argon using a non-consumable electrode into a water-cooled copper vessel [42]. To achieve a uniform composition of the alloy, the ingots underwent 30,000 remelting cycles. The graphite target was machined from an MPG-6 grade graphite block. Silicon (100) platelets served as the substrates for the coating process.

The substrates underwent an initial ultrasonic treatment in a solution of ethanol and acetone (mixed in a 50:50 ratio) and were subsequently dried. The work chamber was pumped to a residual pressure of 1.3 × 10^−3^ Pa and then filled with argon gas through a flow meter at a specified pressure. Prior to the deposition of the coatings, both the substrates and targets underwent sputter etching using Ar ions. The substrates were etched at a negative bias voltage of 500 V for 15 min to eliminate surface contaminants and enhance magnetron discharge stability. The coatings were then deposited onto the substrates with a negative bias voltage of 50 V applied, while the substrates were pre-heated to 400 °C. The magnetrons were operated in the DC regime during the coating process, with a consistent deposition time of 60 min for all samples. The parameters for the TiZrMo target remained constant to ensure an appropriate coating deposition rate. However, for the graphite target, the sputtering current ranged from 150 to 300 mA. A set of TiZrMoC samples was created, and additional details regarding the coating deposition parameters are indicated in Table 1.

### 2.2. Characterization of TiZrMoC Coatings

The morphology and thickness of the coatings were analyzed using a scanning electron microscope, specifically the JEOL JSM 7600F model (Tokyo, Japan). A metallographic process was employed to prepare the samples for cross-sectional viewing. This process involved grinding the samples with SiC abrasive papers, down to 1500 grit, followed by polishing with diamond colloidal suspensions. An energy dispersive spectroscopy system with an X-max 50 mm^2^ detector evaluated elemental composition and mapping. The constituent elements were quantified by averaging the measurements from five different regions.

The elemental composition of the coatings at various depths was analyzed using secondary ion mass spectrometry with a Hiden SIMS workstation (Warrington, UK). This setup included an IG20 ion gun and a maxim HAL7 quadrupole mass analyzer. For the sputtering process, an O_2_^+^ ion beam with an energy of 5 keV and a current of 250 nA was employed.

The crystal structure of the coatings deposited was analyzed using X-ray diffraction with a Panalytical Empyrean X-ray diffractometer (Almelo, The Netherlands). The XRD analysis was conducted in a θ–2θ configuration with Cu K_α_ radiation (*λ* = 0.1541 nm). The X-ray diffraction analysis was performed with the X-ray source operating at 40 kV and 35 mA. The θ–2θ scan range was set between 20° and 100°, with a scan rate of 2°/min and a step size of 0.015°.

The chemical bonding status and chemical composition were analyzed using X-ray photoelectron spectroscopy with a UHV-Analysis System ES-2401 (Berlin, Germany). The XPS analysis utilized Mg Kα radiation with an energy of 1253.6 eV. The base pressure in the sublimation chamber was maintained at less than 10^−6^ Pa. Before the XPS analysis, the coating surface underwent argon etching with an incident energy of 1.5 keV and a current density of 11 μA/cm^2^. During the XPS analysis, the spectra were acquired at a constant pass energy of 20 eV. The Au 4f_7/2_ and Cu 2p_3/2_ peaks with binding energy at 84.0 ± 0.05 eV and 932.66 ± 0.05 eV were used as references, respectively.

The microstructural analysis was conducted using transmission electron microscopy on the JEOL JEM ARM 200CF apparatus (Tokyo, Japan). Images of the sample microstructure were captured at an acceleration voltage of 200 kV in bright-field (BF) high-resolution (HR) TEM, atomic resolution (AR) TEM, and high-angle annular dark-field (HAADF) STEM modes. The sample was thinned to less than 50 nm for TEM analysis using the Nanoanalytik Zeiss Auriga 60 high-resolution focused ion beam scanning electron microscope (FIB-SEM, Carl Zeiss Microscopy GmbH, Jena, Germany) at 30 kV. The final polishing was performed using a beam of 240 pA at 5 kV. Energy Dispersive X-ray Spectroscopy analysis utilized a large-angle JEOL JED-2300T CENTURIO SDD (silicon drift, Tokyo, Japan) detector with a solid angle of up to 0.98 sr and a detection area of 100 mm^2^. For EELS STEM characterization, the GIF Quantum ER imaging filter (Gatan, Pleasanton, CA, USA) was employed. EELS spectral analysis (EELS SI) was conducted in DualEELS mode with a dispersion of 0.5 eV/ch.

The Knoop hardness of the coatings was assessed via indentation tests using the Micromet 2103 microhardness tester (Bluehler Ltd., Tokyo, Japan/Leinfelden-Echterdingen, Germany) with a 100 mN load. This specific load was selected to ensure significant plastic deformation of the coating while minimizing the impact of the substrate material. The uncertainties associated with the hardness measurements were maintained within 5%. Ten indentations were made on each sample, and the average values were recorded and reported.

The friction coefficient for all samples was determined using a pin-on-disk friction machine following the ASTM-G99 [43] procedure for wear testing. The tests were conducted at room temperature without lubrication in ambient air with a relative humidity of approximately 50%. The test setup involved a vertically loaded 4 mm diameter silicon nitride ball functioning as a pin sliding against the rotating silicon plate with coatings. The normal force applied on the Si_3_N_4_ ball was 0.5 N, with a specimen rotation speed of 60 rpm. The linear sliding rate was approximately 0.02 m/s, and the tests lasted for 20 min. During the experiments, the friction force was continuously recorded, allowing for the calculation of the friction coefficient.

The wear test of S3 was conducted, utilizing the ball-on-disk sliding method with CSM Instruments under dry conditions at a room temperature of 30 °C and a relative humidity of 40%. A tungsten carbide (WC) ball with a diameter of 5 mm served as the counter-sample. In this test, the WC ball applied a load of 0.75 N and slid against the specimen’s surface until the cycle number reached 30,000. The estimation of the friction coefficient and wear factor was carried out without removing wear debris from the friction pair. The wear tracks were examined using SEM, employing secondary electron imaging to visualize surface morphology. Additionally, the elemental composition of the wear track was determined using Energy Dispersive X-ray Spectroscopy Mapping. The wear rate (*W_R_*) was calculated utilizing the following equation:

(1) $$W_{R}=\frac{h(3h^{2}+4b^{2})2\pi r}{6bL_{n}S},$$

where *h*, *b*, and *r* are the wear track’s depth, width, and radius, respectively, *L_n_* is the normal load, and *S* is the total sliding distance.

### 2.3. Computational Procedure

To investigate the mixing behavior of the random (Ti_0.5_Zr_0.5_)_1−x_Mo_x_C, (Ti_0.5_Mo_0.5_)_1−x_Zr_x_C and (Zr_0.5_Mo_0.5_)_1−x_Ti_x_C quaternary solid solutions (alloys), we used fcc Special Quasi Random Structures (SQS) generated in Ref. [44] for TM alloys with compositions *x* = 1/3 (24 atoms) and 1/2 (32 atoms). The 16-atom SQSs for fcc binary random alloys with equiatomic composition [45] were used to model the random Ti_0.5_Zr_0.5_C, Ti_0.5_Mo_0.5_C, and Zr_0.5_Mo_0.5_C alloys (in further denoted as TiZrC, TiMoC, and ZrMoC, respectively). The solid solutions’ SQSs for the quaternary carbide alloys consisted of 48 and 64 atoms, while the ternary carbide alloys contained 32 atoms, considering that carbon creates an inactive sub-lattice.

The generalized gradient approximation (GGA) for the exchange-correlation energy and potential, as detailed in the referenced paper [46], was employed. The plane-wave cut-off energy utilized in the calculations was set to 490 eV. Various Monkhorst-Pack k-point meshes were employed, depending on the size of the unit cell: (2 2 2) for the 64-atom supercells, (2 2 4) for the 48-atom supercell, (2 3 4) for the 32-atom supercells, and (12 12 12) for 2-atom cells. The Gaussian smearing scheme with a smearing parameter of 0.272 eV was employed for Brillouin-zone integration. The geometry optimization of all initial structures involved relaxing the cell vectors and atomic positions using the Broyden–Fletcher–Goldfarb–Shanno method [47]. The relaxation process was terminated when the atomic forces were below 25.7 meV/Å, stress was less than 0.05 GPa, and the total energy exhibited a variation of no more than 0.001 eV during the structural optimization. The symmetry of the relaxed SQSs was confirmed using the ISOTROPY code [48]. We found that they had the Fm-3m symmetry with tolerances of 0.005*a_0_* (quaternary alloys) and 0.01*a_0_* (ternary alloys), where *a_0_* = (8·*V*/*N_a_*)^1/3^, and *V* and *N_a_* were the volume and number of atoms in the relaxed supercell.

The ElaStic code [49] was utilized to compute the elastic constants and determine the Hill bulk (*B*), shear (*G*), Young (*E*) moduli, and Poisson’s ratio (*ν*). The empirical formulas are outlined in the references [50,51] were employed to estimate the fracture toughness (*K_IC_*) and Vickers hardness (*H_V_*). The mixing energy (*E_MIX_*) of the carbide systems was calculated as follows:

*E_MIX_*(Ti_x_Zr_y_Mo_z_C) = [*E_T_*(Ti_x_Zr_y_Mo_z_C) − *x*·*E_T_*(TiC) − *y*·*E_T_*(ZrC) − *z*·*E_T_*(MoC)]/(*x* + *y* + *z*), (2)

In the calculation, *E_T_* represents the total energy of the SQSs, NaCl-type TiC, ZrC, and WC-type MoC. Equation (2) considers the hexagonal polytype of MoC due to the potential formation of cubic molybdenum carbide under conditions involving a deficit of atoms in the carbon sub-lattice or at extremely high temperatures [52]. Additionally, it was demonstrated that the stability of the cubic solid solutions derived from MoC was ensured as long as the concentration of Mo atoms *z* (refer to Equation (1)) remained below 0.75 [52]. Hence, the analysis focused solely on the composition of the cubic Ti_x_Zr_y_Mo_z_C alloys for *z* < 0.75.

## 3. Results and Discussion

### 3.1. Nanostructure and Chemical Bonding

**Figure 1:**
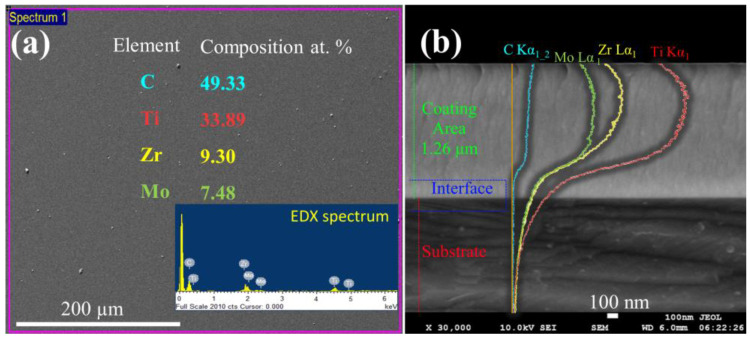
Surface (**a**) and cross-section SEM image (**b**) with EDX spectra and line scan obtained from EDX analysis of the TiZrMoC S3.

The TiZrMoC samples fabricated through magnetron sputtering exhibit uniformity and compactness, with no apparent defects such as voids or visible pores. Due to the consistent deposition regime of the TiZrMo target, no substantial variance in or composition was detected from S1 to S4. Additional detailed analysis will be presented for S3 as it is deemed representative and the most promising in terms of tribological and mechanical properties (refer to Section 3.2). The morphology of S3 is illustrated through the top view and cross-sectional scanning electron microscope (SEM) micrographs in Figure 1. The estimated thickness is around 1.26 µm, with similar morphologies observed in the other coatings obtained. These columnar structures are associated with the utilization of a DC magnetron source, known for generating denser coatings attributed to the high-energy ion bombardment. The X-ray diffraction (XRD) data confirm that the columns are well-defined and comprise small crystallites. Analysis of the energy-dispersive X-ray spectroscopy (EDX) data from the surface reveals a composition consisting of 49.3 at.% of C, 33.9 at.% of Ti, 9.3 at.% of Zr, and 7.5 at.% of Mo.

**Figure 2:**
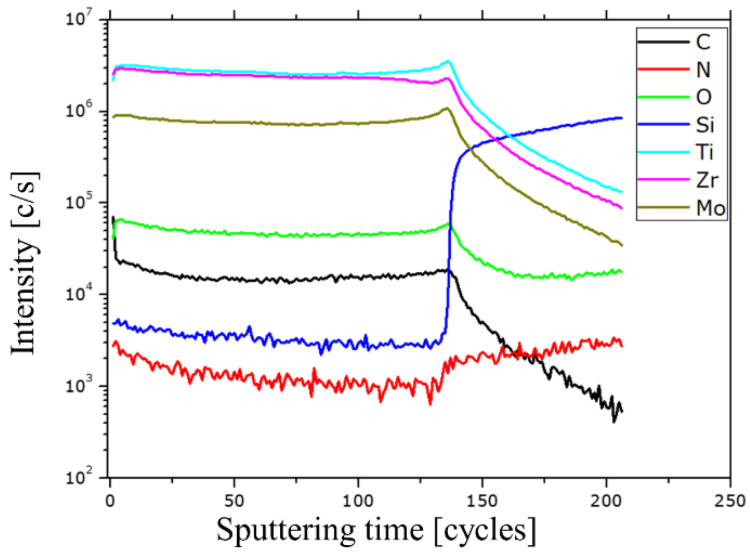
SIMS depth element profiles of TiZrMoC S3 coating on a logarithmic scale. Raster size 400 μm × 500 μm. Electronic gate 3.0%.

The secondary ion mass spectrometry (SIMS) analysis indicated variations in intensity between the metal elements and their compounds (Ti, Zr, and Mo). Zr and Mo displayed consistent signal intensities across the metal and its carbide compositions. Furthermore, the M^+^ signals exhibited higher intensities attributed to the matrix effect compared to the metal-carbide (MC^+^) or metal-oxide (MO^+^) signals. The SIMS depth profile examination involved positive ions with the respective analyzed masses of ^12^C, ^14^N, ^16^O, ^30^Si, ^49^Ti, ^90^Zr, and ^96^Mo. Figure 2 presents the acquired SIMS depth profiles depicting the elemental distribution within the TiZrMoC coating.

The data obtained can be leveraged to quantify the composition of the deposited mixed carbide layer. To facilitate this, secondary ion current ratio plots have been generated based on the raw data from Figure 2. Further details and information can be found in the Supporting Information (Figures S1 and S2).

**Figure 3:**
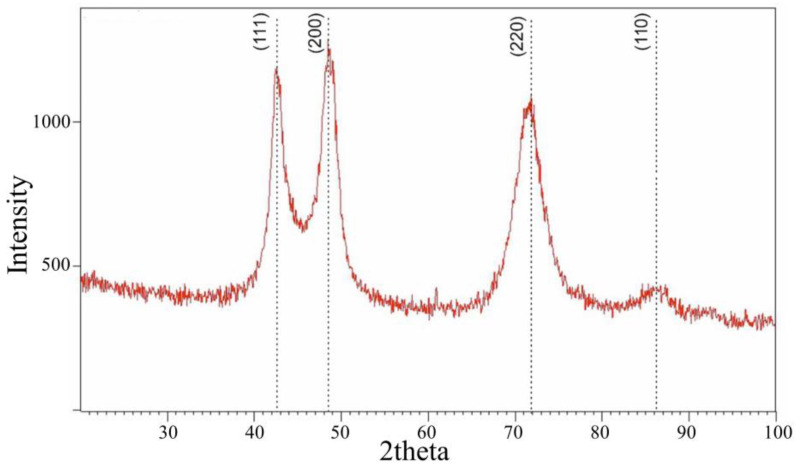
XRD pattern of TiZrMoC S3. Black dotted lines show the location of TiC peaks.

In Figure 3, the typical X-ray diffraction (XRD) pattern of the TiZrMoC S2 coating is displayed. The XRD assessment revealed the presence of three diffraction peaks: (111), (200), and (220). These peak positions align with a face-centered cubic (fcc) structure characteristic of fcc-TiC. Additionally, no Bragg peaks corresponding to ZrC, MoC, or solid solution carbides such as (Ti, Zr)C and (Ti, Mo)C were identified. Therefore, the central component of the TiZrMoC coatings is the titanium-carbide system, specifically featuring a refractory cubic monocarbide. Notably, the phase Ti_2_C is absent in the equilibrium phase diagram, indicating a propensity for carbon ordering at the stoichiometry level. Titanium carbide (TiC_x_) exhibits a broad range of stoichiometry, extending from *x* = 0.47 to 0.98 [7,53,54]. The crystal structure of titanium is interstitial, accommodating carbon atoms within the voids of the titanium lattice. This transition occurs from the hexagonal α-Ti structure with space group P63/mmc to the face-centered cubic δ-TiC structure with space group Fm-3m, where the carbon atoms occupy the octahedral sites within the titanium lattice as the carbon content increases. However, physical vapor deposition techniques, such as magnetron sputtering, represent highly non-equilibrium processes characterized by rapid cooling rates resulting from the immediate condensation of species onto a substrate. Consequently, in such conditions, it is plausible that the α-Ti lattice may be compelled to incorporate more carbon atoms due to the restricted mobility of the deposited particles.

The presence of (111), (200), and (220) planes with comparable intensities remains a topic requiring further elucidation. Various studies attribute this phenomenon to the interplay between strain and surface-free energy within the material. According to Pelleg et al. [55], TiC tends to exhibit growth with a preferred orientation along the (200) plane during its initial stages due to its minimal surface energy. The rationale behind this preferred orientation is primarily rooted in thermodynamic factors, overlooking kinetic influences like ion bombardment, composition, incident ion angle, and energy. Typically, coatings deposited under low-energy conditions (e.g., low temperature, minimal ion bombardment) tend to exhibit a (111) crystallographic texture [56,57].

The observation of a peak displacement toward lower 2theta values (by 0.8°) may be influenced by the presence of Zr and Mo elements. The substitution of smaller atoms for the base atoms in transition metal (TM) carbide solid solutions, such as in (Ti, Mo)C, typically leads to a shift of Bragg reflections toward higher angles due to a reduction in the unit cell size. Given the disparate atomic radii of molybdenum (0.140 nm) and zirconium (0.158 nm) from titanium (0.149 nm), the inclusion of larger zirconium atoms is expected to expand the cell parameters, causing the Bragg reflections to shift toward lower angles. Therefore, despite the lower Zr atom content, their impact outweighs that of Mo atoms, resulting in the diffraction peaks shifting toward lower angles.

The crystal size of the coating, estimated through the Scherrer equation from peak broadening, falls within the nanometer scale (10–12 nm). Despite assuming a single-phase solid solution carbide based on the Vegard Law, the measured lattice constants (*a* = 0.4582) exhibited a shift toward higher values than anticipated positions. Lewin et al. [58] attributed a change in lattice constant to a change in the Fermi level in the titanium carbide grains. The higher electronegativity of carbon compared to titanium within the matrix facilitates a partial electron transfer from titanium to carbon at the grain boundary interface. Consequently, this process lowers the Fermi level, reducing the occupancy of bonding states, which in turn diminishes binding forces and enlarges the lattice constants. The ab initio density functional theory model, designed for metal carbides featuring a NaCl-type structure and VEC of 4 [58], was initially established with data validation from nanocrystalline titanium carbide combined with amorphous carbon. Although this model could be extended to coatings within the zirconium-carbon and molybdenum-carbon systems, it falls short of fully explaining the observed lattice constant shift observed in this study. By incorporating zirconium and molybdenum into the model, a larger lattice constant is anticipated as the grain size decreases. This extension could enhance the insights established by Lewin et al., particularly regarding potential impacts on the electronic structure (e.g., varying electronegativity) while preserving the NaCl-type structure. Notably, Adjaoud et al. propose a positive deviation from the ideal Vegard law through first-principles calculations for various systems, with a specific emphasis on the TiC-ZrC combination [59]. Shifts in lattice constants could also be attributed to lattice distortions, interstitial elements, or induced stress within the material.

The sample was also designated according to the residual stress magnitude using the cos^2^*α*sin^2^*ψ* method [60]. It was revealed that the residual stress of the TiZrMoC sample was −3.58 GPa. The observed value signifies the development of intermediate-level compressive residual stresses. This phenomenon is linked to the substitution of smaller titanium atoms with larger zirconium atoms within the TiN-matrix lattice, leading to notable lattice distortions and the consequential generation of residual stress.

**Figure 4:**
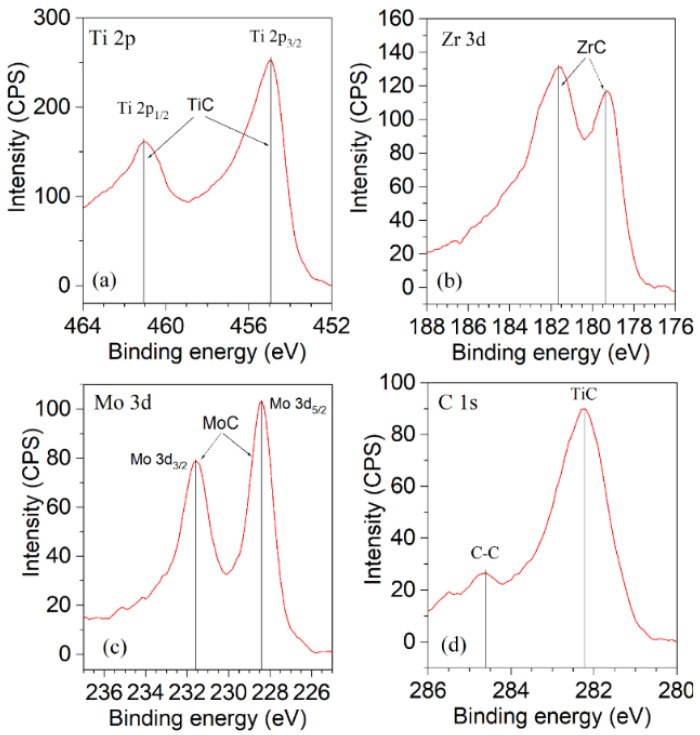
Typical XPS spectra of TiZrMoC S3: (**a**) Ti 2p line, (**b**) Zr 3d line, (**c**) Mo 3d line, (**d**) C 1s line.

Figure 4 displays the typical X-ray photoelectron spectroscopy (XPS) spectra acquired for sample S3. By analyzing the spectra peaks, we identified the presence of various bonds, as summarized in Table 2. The results suggest the formation of bonds such as Ti-C, Zr-C, Mo-C, and C-C within the samples, while no Ti-Ti, Mo-Mo, or Zr-Zr bonds were detected. This indicates a predominance of mixed carbide phases over pure metal phases in the coatings.

**Table 2:** Details of peak identification in XPS spectra from Figure 4.

XPS Spectrum	Peak Position [eV]	Identification	Reference Data [eV]
Ti 2p3/2	454.9	TiC	455.05 [61], 455.0 [62]
Ti 2p1/2	461.1	TiC	461.3 [61], 461.0 [62]
Zr 3d5/2	179.3	ZrC	179.1 [61], 179.6 [63]
	181.65	ZrC	181.5 [61]
Mo 3d3/2	231.57	MoC	231.4–232.1 [64,65,66]
Mo 3d5/2	228.4	MoC	228.2–228.8 [64,65,66]
C 1s	282.2	TiC	281.9 [67], 282.2 [68]
C 1s	284.6	C-C	284.5 [68], 284.6 [67]

The discrepancy in composition ratios between the XPS and EDX data can be attributed to the inherent sensitivity differences in these analytical techniques. XPS, with its high sensitivity of approximately 10^−3^ at.%, is capable of detecting even minor elemental variations within the surface layers. In contrast, EDX, while useful for bulk analysis, has a significantly lower sensitivity, often in the order of 1%. As a result, XPS can provide a more detailed and accurate representation of the surface composition, including potential surface enrichment or depletion of certain elements. This sensitivity difference can lead to variations in the calculated composition ratios, especially when dealing with complex materials or thin films [69].

The microstructure of certain ternary carbides (TM1/TM2)C, where TM1 and TM2 stand for the transition metals analyzed in this study, has been explored in earlier research [7,70]. Authors [7] examined TiMoC coatings and found that between 20.4 to 80.8 at.% Mo, the coatings were solid mixes of Mo within TiC. Similarly, a prior investigation [70] identified a (Ti, Zr)C solid mix in Ti_1-*x*_Zr*_x_*C (*x* = 0.25, 0.5 and 0.75) coatings using methods like X-ray diffraction, X-ray photoelectron spectroscopy, and first-principles calculations. These studies hint that the coatings probably feature a nanocomposite structure, possibly displaying a setup resembling nc-Ti_1-x_Zr_x_C_y_/a-C.

The analysis of XRD and XPS data in our research revealed the creation of a solid solution (Ti, Zr, Mo)C, illustrating the coating structure as nanocrystals of this solid solution surrounded by the a-C phase. This supports our conclusions and backs the idea of nanocomposite formation, which may play a crucial role in enhancing the mechanical and tribological characteristics of the coatings.

Based on XPS data, the TiZrMoC S3 coating, which exhibited the highest hardness of approximately 34 GPa (as elaborated further below), possessed the composition Ti_60_Zr_25_Mo_15_/C. Throughout our study, the sputtering parameters remained consistent across all experiments, ensuring uniformity in the composition of TM components across all coatings.

**Figure 5:**
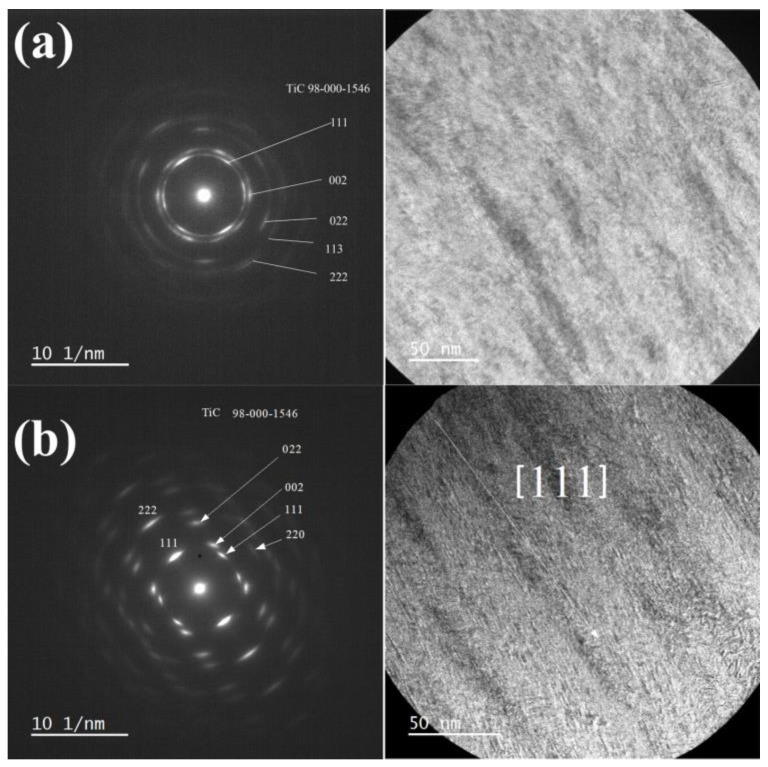
Transmission electron microscopy characterization of TiZrMoC S3, recorded from the area: (**a**) close to the substrate; (**b**) close to the surface. Left image: selected area diffraction and identification of diffraction rings attributed to a crystalline structure with fcc; Right image: symmetryTEM image of the coating cross-section. Please note that diffraction is non-uniform, indicating preferential growth.

Figure 5 illustrates low-magnification transmission electron microscopy (TEM) images and the associated electron diffraction outcomes of the TiZrMoC coating (S3) in two regions: proximal to the substrate (a) and the surface (b). Both selected-area electron diffraction (SAED) patterns exhibit concentric rings of varying intensity, signifying a textured growth pattern in the coating. Analysis of the SAED pattern in Figure 5a enabled the determination of interplanar distances measuring 0.253 nm, 0.221 nm, 0.157 nm, 0.134 nm, and 0.125 nm. Although measured values of interplane spaces are a little higher than that reported in PDF [089-3828], these values can be assigned to (111), (002), (022), (113), and (222) crystal planes of TiC, exhibiting cubic crystal structure with space group 225. The increase in interplane spacings can be elucidated by incorporating Zr atoms into the TiC crystal structure.

The coating displays a columnar growth perpendicular to the sample surface. In the bottom area, the crystals that belong to the coating are smaller and more disorientated, Figure 5a. The upper part of the coating is highly textured, which follows from Figure 5b.

**Figure 6:**
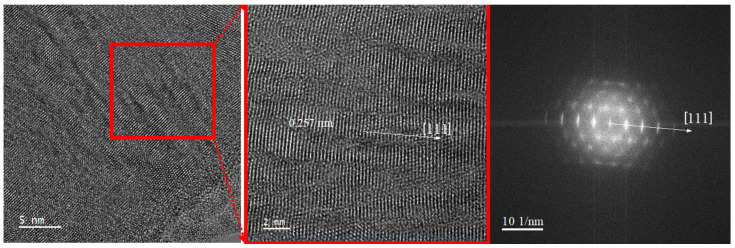
A high-resolution TEM image of the TiZrMoC S3 cross-section close to the surface area and corresponding FTT pattern demonstrates the strong texture with the elongated nanograins.

Figure 6 shows the high magnification TEM (HRTEM) images and corresponding Fast Fourier Transform (FFT) pattern of TiZrMoC coating from an area close to the surface. The coating exhibits a strong crystallographic texture in [111] direction and is made up of columnar grains with diameters from 2 nm to 4 nm (red frame in Figure 6).

**Figure 7:**
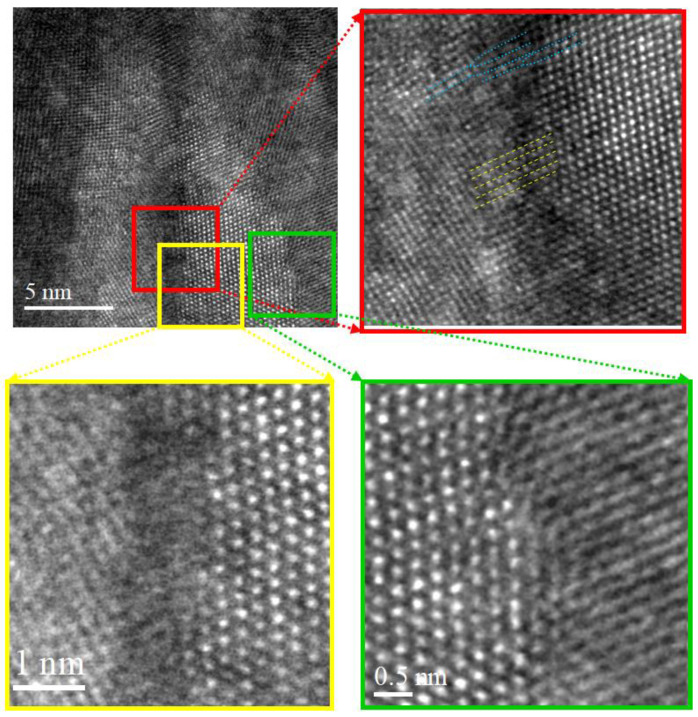
HAADF STEM image of the TiZrMoC S3 cross-section (top of the coating), demonstrating the boundary between adjacent elongated nanograins.

The detailed boundary image between adjacent elongated nanograins recorded by HAADF STEM (Z-contrast imaging) is presented in Figure 7. Images such as those in Figure 6 and Figure 7 were recorded from multiple areas (minimum 10 fields of view across three different samples) to ensure the observed features are reproducible and not artifacts of a specific region. A typical image shows various types of grain boundaries in the coating. As can be seen from these images, grain boundaries are highly defective (blue lines in Figure 7 red rectangle insert) [71,72]. The grain boundary characteristics described here were consistently observed in more than 80% of the tested regions across the examined samples, supporting the reproducibility of the defect distribution. However, there are parts of the boundary where the alignment between grains is perfectly adjusted (yellow lines). The nanocrystal is separated from the neighboring crystal by a layer of the amorphous phase that is displayed in the HAADF STEM image as a darker strip, Figure 7 (yellow rectangle insert), with thickness ranging from 0.5 nm to 1.5 nm. EDX point analysis showed that the amorphous grain boundary layer was enriched by carbon (55.8–68.3 at.%). In the meantime, the content of carbon inside the crystallites ranged from 42.3 at.% up to 48.5 at.% across coating depth. Based on this, it can be assumed that the amorphous layer separating nanocrystallites is a:C.

However, the majority of grain boundaries were of TiC-TiC type. An example TiC-TiC grain boundary, as was revealed by the HAADF study, is presented in Figure 7 (green rectangle insert).

**Figure 8:**
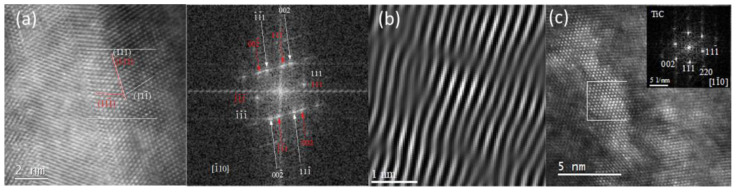
HAADF STEM image from the top of the TiZrMoC S3 coating (**a**) at atomic resolution along the [$\bar{1}$10] zone axis showing (111) a twin plane in the structure of the grain and Inverse FFT (IFFT) image (**b**) of deformed lattice fringes and several misfit dislocations inside the TiZrMoC grains. (**c**) Atomically resolved HAADF-STEM image for [110] the oriented TiC elongated nanocrystal and FFT pattern of the selected area (white square).

HRTEM analysis revealed that TiZrMoC nanograins in the lower part of the coating exhibited a high density of defects, including nanotwins and stacking faults. For instance, a twin in a TiZrMoC grain with a (111) twin plane is visible in the HAADF STEM image and the corresponding FFT pattern (see Figure 8a) [73]. This image was obtained from one of the twinned regions in a TiC nanocrystal oriented along the [−110] zone axis, illustrating nanotwins with a (111) twinning plane.

**Figure 9:**
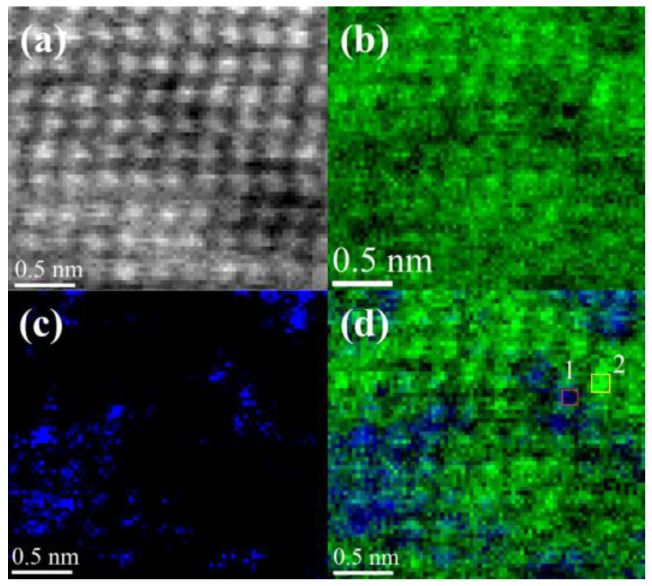
Atomic columnar structure of the TiZrMoC S3 crystal oriented along the <110> direction: (**a**) ADF image at atomic resolution of analyzed area; (**b**) EELS map at atomic resolution of Ti-L_2,3_ edge recorded from area in (**a**); (**c**) EELS map of Zr -M_4,5_ edge recorded from area in (**a**); (**d**) overlay of Zr-M_4,5_ and Ti-L_2,3_ EELS maps.

The upper portion of the coating consists of columnar grains with much fewer defects. Twinning was not observed there, and except for misfit dislocations, the warping of lattice fringes was found inside the grains, as is imaged using inverse FFT images (IFFT) Figure 8b. Figure 8c presents a detailed atomically resolved HAADF STEM image of an elongated nanocrystal. This nanocrystal, approximately 3 nm in diameter and 12–14 nm in length, aligns well with the crystallite size predicted by XRD calculations. The crystal’s orientation along the [110] zone axis is confirmed by the corresponding FFT pattern (Figure 8c inset). It is supposed that local compositional changes in TiZrMoC grains can cause dislocations and warping of lattice fringes. This is confirmed by Figure 9a–d.

The ADF-STEM image in Figure 9a displays the distribution of atom columns containing Ti, Zr, and Mo in the cubic structure of the nanocrystallite oriented along <110> direction. Compositional changes in TiZrMoC grains are evident from Figure 9b,c, where atomic maps of Ti and Zr using Ti-L_2,3_ and Zr-M_4,5_ edges are recorded using the EELS method from the same area [74,75]. Figure 9d presents the superposition of Zr-M_4,5_ and Ti-L_2,3_ EELS maps. Changes in chemical modulations are evident from this image. The differences in chemical composition are noticeable also from quantitative EELS measurements acquired from atomic columns 1 (Zr = 10.8 ± 0.5 at.%, Mo = 8.9 ± 0.4 at.%, C = 47 ± 2 at.%, and Ti = 33.7 ± 1.7 at.%) and 2 (Zr = 7.3 ± 0.4 at.%, Mo = 5.4 ± 0.3 at.%, C = 45 ± 2 at.%, and Ti = 43± 2 at.%) regions outlined with a red and yellow frame in Figure 9d.

### 3.2. Tribological and Mechanical Properties

**Figure 10:**
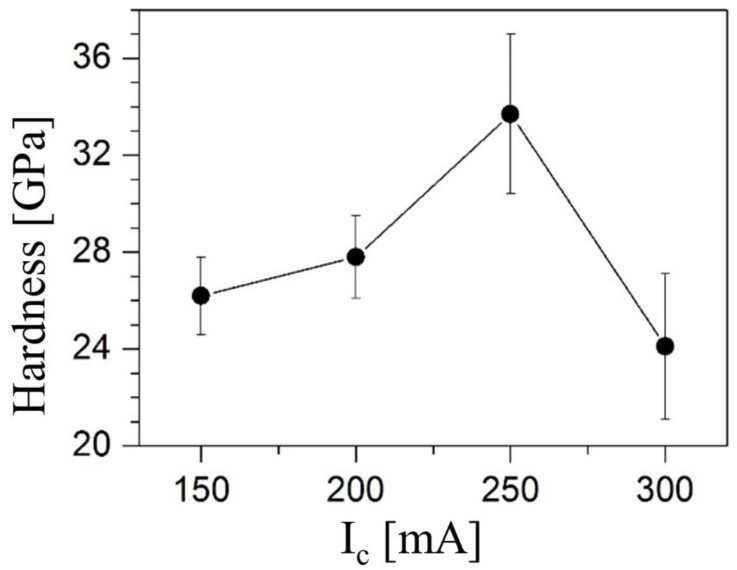
Knoop hardness (*HK*) as a function of the graphite target (*I_C_*) current.

Figure 10 presents the relationship between the Knoop hardness of the deposited coatings and the current at the graphite target during sputtering. The hardness exhibited a continuous rise with increasing sputtering current at the graphite target, peaking at approximately 35 GPa at *I_C_* = 250 mA. Table 3 provides a comprehensive overview of the hardness values for the Ti-Zr-Mo-C system compared to other similar systems, highlighting the methods used and referencing the pertinent literature.

The Ti-Zr-Mo-C coating system exhibits superior hardness (24–34 GPa) compared to other ceramic systems due to its optimized composition and sputtering parameters. The inclusion of Mo enhances hardness by increasing elastic moduli and reducing defect density, while lattice distortion from interstitial carbon atoms further strengthens the coating. This system outperforms traditional TiC, ZrC, and TiMoC coatings, making it highly suitable for applications requiring exceptional wear resistance and durability. The key factors contributing to its performance optimization are its valence electron concentration, minimized cell volume disparity, and careful control of deposition conditions.

**Table 3:** Comparison of hardness values with known ceramic binary systems.

System	H [GPa]	Method	Refs.
Ti-Zr-Mo-C	24–34	Coatings (DC magnetron)	This work
Ti-Nb-C	20–25	Bulk (hot press)	Fides et al. [76]
Ti-Nb-C	26–29	Coatings (laser cladding)	Sun et al. [77]
Ti-Mo-C	8–10	Coatings (RF magnetron)	Koutzaki et al. [7]
Ti-W-C	15–26	Coatings (RF magnetron)	Koutzaki et al. [7]
Ti-Zr-C	26–33	Coatings (DC magnetron)	Pogrebnjak et al. [70]
Ti-Zr-C	14–34	Coatings (DC magnetron)	Rodríguez-Hernández et al. [78]

The hardness of TiZrMoC is slightly higher than that reported for TiC (16–18 GPa [79]), ZrC (25 GPa [80]), MoC (20 GPa [81]), TiMoC (10 GPa [7,82]), and TiZrC (33 GPa [70]) coatings. However, the values for Ti-Zr-C and TiZrMoC are very similar and likely fall within the experimental uncertainty. Further increasing the sputtering current resulted in a decrease in hardness to around 24 GPa.

The reduced hardness observed in TiMoC coatings in previous studies [7,82] was linked to the significant presence of amorphous material and voids between the grains of the (Ti, Mo)C solid solution. The significantly elevated hardness observed in TiZrC coatings in a prior study [70] was hypothesized to stem from the occupation of interstitial sites by carbon atoms. Consequently, lattice distortion occurred, hindering dislocation motion and initiating the process of strengthening the coating. Marupalli et al. [21] conducted a study on the nanohardness of a Ti_52_Zr_40_Mo_8_ coating deposited via a DC/RF magnetron sputtering system onto a Si (100) substrate. Their findings indicated that the as-deposited coating exhibited a hardness of 7.8 GPa, a value primarily ascribed to the predominantly amorphous nature of the coating was established.

The graphite target current significantly influences the hardness of TiZrMoC samples. While all samples share similar structural and compositional features, distinct hardness trends emerge. S1 (150 mA) and S2 (200 mA) exhibit progressively increasing hardness due to incremental carbon incorporation, which strengthens the coating through lattice distortion and enhanced grain boundary cohesion. However, at lower currents, the carbon content is insufficient to achieve the optimal balance of nanocrystalline and amorphous phases. Conversely, S4 (300 mA) demonstrates a drop in hardness to approximately 24 GPa, likely due to excessive carbon promoting the formation of softer graphitic phases and introducing voids or weak interfaces. S3 (250 mA) achieves the highest hardness of 35 GPa, benefiting from its ideal carbon content, which facilitates a finely tuned microstructure with nanocrystalline grains, stacking faults, and a controlled amount of amorphous carbon. This unique balance optimizes hardness by enhancing load distribution, reducing defect motion, and maintaining structural integrity under stress.

**Figure 11:**
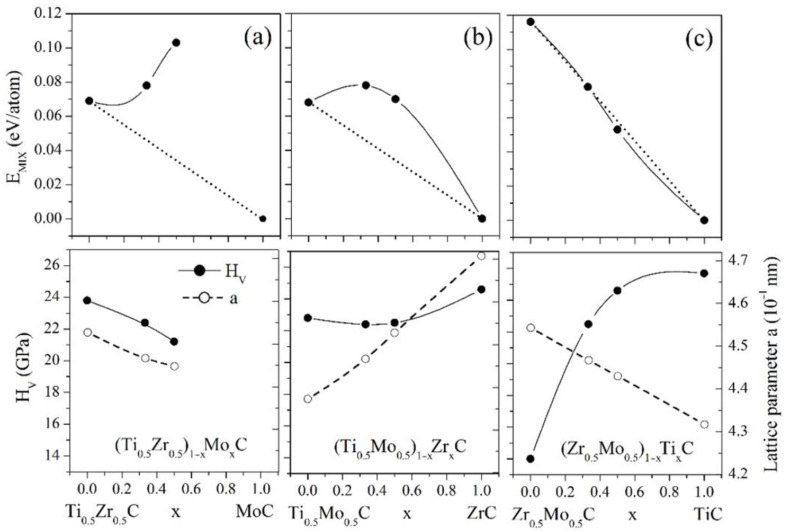
Mixing energy (*E_MIX_*), Vickers hardness (*H_V_*), and lattice parameter (*a*) for the TiZrC-MoC (**a**) TiMoC-ZrC (**b**) and ZrMoC-TiC (**c**) ternary solid solutions. Data shown are from the computational study.

To gain deeper insights into the characteristics of the deposited coatings, we conducted first-principles calculations on random TiZrMoC alloys. Our focus was primarily on exploring the impact of substituting atoms in the ternary carbide alloys TiZrC, TiMoC, and ZrMoC with atoms from different compounds. Figure 11 depicts the compositional variations in the mixing energy, Vickers hardness, and lattice parameter for three sets of random quaternary alloys within the TiZrMoC system. Due to the factors mentioned previously, we refrained from interpolating the calculated values for the molybdenum-enriched alloys in the 0.5 < *z* < 1 range. Notably, all the ternary alloys exhibit positive mixing energies, suggesting that these materials are prone to decomposition at specific annealing temperatures. An introduction of Mo and Zr atoms into TiZrC and TiMoC, respectively, leads to the formation of the quaternary TiZrC-MoC and TiMoC-ZrC solid solutions for which the *E_MIX_*(*x*) dependences have a positive deviation from linearity (relative to the dotted lines in Figure 11). That means the parent carbides of these systems are insoluble. On the contrary, ZrMoC and TiC are soluble and form ideal solid solutions since the mixing energies of the ZrMoC-TiC quaternary are slightly below the linear dependence of *E_MIX_*(*x*) (see Figure 11c).

The lattice parameter increases with *x* in (TiMo)_1−x_Zr_x_C and decreases with increasing *x* in (TiZr)_1−x_Mo_x_C and (ZrMo)_1−x_Ti_x_C. This finding and the *a*(*x*) dependences presented in Figure 11 show that the peaks in the XRD pattern for the quaternary (TiZrMo)C alloys will be located at lower values of 2θ than those for TiC in agreement with the XRD results (Figure 3).

Figure 11 and Table 4 present the calculated mechanical properties of the carbide alloys. MoC and TiMoC exhibit the highest elastic moduli among all carbides. Additionally, the values of bulk modulus (*B*), shear modulus (*G*), and Young’s modulus (*E*) for all quaternary carbides surpass those of TiC, ZrC, and TiZrC. The incorporation of molybdenum in ternary carbide alloys enhances their elastic moduli. It is anticipated that all carbide structures, except for ZrMoC, will demonstrate brittle behavior, with ZrMoC expected to exhibit ductile characteristics. Among the carbides, zirconium carbides exhibit the lowest fracture toughness. Moreover, the fracture toughness is observed to rise with increased molybdenum content.

Table 4 displays the structural parameters, mixing energies, Hill elastic moduli, Poisson ratio, *B*/*G* ratio, Vickers hardness, and fracture toughness of binary, ternary, and quaternary carbide alloys within the TiZrMoC system. The calculated lattice parameter, *a*, for B1-TiC and B1-ZrC (space group Fm-3m) and the *a* and *c* parameters for MoC (space group P-6m2) were 4.317 Å (4.317 Å, PDF [089-3828], 4.328 Å PDF [071-0298]), 4.702 Å (4.692 Å PDF [065-0973]), and *a* = 2.926 Å, *c* = 2.84 Å (reference values 2.901 Å and 2.786 Å, respectively, PDF [029-1129]), respectively (in parenthesis the corresponding experimental lattice parameters are shown). The computed lattice parameters are marginally larger than the observed values due to the tendency of the GGA approximation to slightly overestimate structural parameters. In a prior publication [70], we contrasted the computed mechanical properties of TiC and ZrC, noting their alignment with results from other experimental and theoretical investigations. Concerning solid solutions, our examination revealed only a first-principles investigation of the Ti_0.5_Mo_0.5_C alloy [83]. The values of *B* = 295, 308, *G* = 180, 200, *E* = 442, 481, and *H_V_* = 20.3, 23.8 (in GPa), where the first values are for SQS and the second values are for the ordered cubic structure C#3 obtained in that study, are comparable to those listed in Table 4 for this alloy. The properties determined in our study exhibit a strong agreement with findings from various experimental and theoretical studies, thus significantly validating the selected computational methodologies.

Next, we will examine how the composition influences the hardness of the ternary carbide alloys. One can see from Figure 11 that for (TiMo)_1−x_Zr_x_C and (TiZr)_1−x_Mo_x_C, the Vickers hardness decreases as *x* increases, whereas it increases in (ZrMo)_1−x_Ti_x_C with increasing titanium content. In Reference [13], it was discovered that the highest hardness is anticipated in solid solutions derived from TM carbides when their valence electron concentration approaches 8.5 and there is a minimal disparity in cell volume between the parent carbides. This principle was confirmed through various carbide alloys, such as TiC-MoC solid solutions [52]. This principle also holds for our quaternary carbide alloys. Specifically, replacing Zr atoms with Mo atoms in Ti_0.5_Zr_0.5_C (VEC = 8) results in the formation of Ti_0.5_Zr_0.25_Mo_0.25_C alloys (VEC = 8.5) and a subsequent enhancement in hardness. However, the enhancement in hardness was not observed for the Ti_0.25_Zr_0.50_Mo_0.25_C alloys (VEC = 8.5) when the Ti atoms were substituted by Mo ones. That can be explained by the fact that in the first case, the difference in the cell volumes of the parent carbides was less than in the second case. The composition of the deposited coatings is close to the composition of the Ti_0.5_Zr_0.25_Mo_0.25_C alloy considered here. This prompts us to assume that the observed hardness enhancement of the deposited TiZrMoC coatings compared to the TiZrC coatings [70] can be related to the mechanism described above.

**Figure 12:**
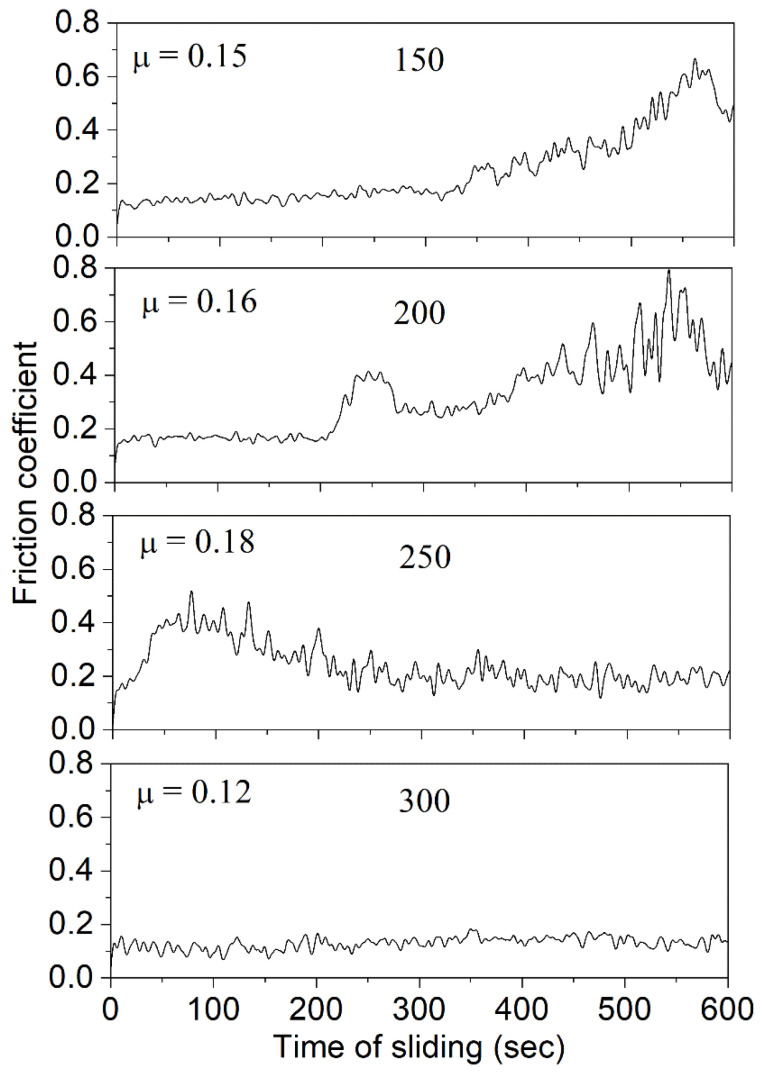
Dependence of the friction coefficient on the sliding time for TiZrMoC coatings. Numbers in graphs denote the values of sputtering current at a graphite target and the average friction coefficient *µ*.

Figure 12 illustrates the relationship between the friction coefficient and sliding time for TiZrMoC coatings. The numbers within the graphs represent the values of the sputtering current at the graphite target and the average friction coefficient (*µ*). The specimen deposited with the maximum sputtering current used at the graphite target (300 mA) exhibits consistently low friction coefficients, hovering around or slightly above 0.1 throughout the sliding time. Similarly, the specimen deposited at a lower sputtering current of the graphite target (250 mA, S3) also demonstrates a low friction coefficient, except during the initial run-in period. In contrast, specimens deposited with the lowest sputtering currents initially display a minimal friction coefficient at the beginning of sliding. However, following a stable initial period, the friction coefficients of these specimens increase. The sputtering current impacts the number of sputtered species, with higher currents leading to more sputtered (and subsequently condensed) species. Therefore, the low friction coefficient observed in sample S4, prepared with 300 mA, can be attributed to a relatively high carbon content in the coating structure. As the sputtering current decreases, the carbon content also decreases. Consequently, the removal of graphite layers surrounding nanocrystals of (TiZrMo)C solid solution, which acts as a solid lubricant, from the contacting surfaces leads to an increase in friction.

The observed variation in tribological behavior is influenced by several factors. Increased sputtering current (S1 to S4) enhances carbon incorporation, promoting a lubricating graphite-like amorphous carbon layer. This layer reduces friction by minimizing contact between asperities. S4, with the highest carbon content, benefits most, while S1 and S2 suffer from insufficient lubrication and increased surface roughness due to wear. Additionally, the presence of minor oxycarbides or other byproducts at lower currents may further contribute to friction. In summary, the optimal balance of carbon content, as achieved in S3, leads to a superior combination of mechanical properties and tribological performance.

**Figure 13:**
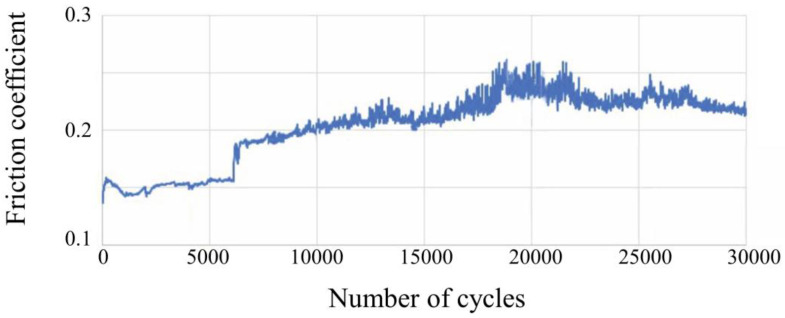
Dependence of the friction coefficient on the number of sliding cycles and detailed test parameters for TiZrMoC S3.

The graph in Figure 13 displays the friction coefficient against sliding distance obtained from wear tests conducted on TiZrMoC coating S3. (The test details are given in Table 5). By analyzing the friction coefficient graph over 3000 cycles, it is observed that the coating’s friction coefficient initiates at 0.135, gradually rises to 0.154 by 6000 cycles, experiences a sudden increase to 0.180, and then steadily grows to around 0.25 within the range of 6000 to 19,000 cycles. Subsequently, the friction coefficient decreases until 20,000 cycles before stabilizing between 0.22 and 0.23 until the conclusion of the experiment at 30,000 cycles.

During the initial 6000 cycles, slight fluctuations in the coefficient of friction graph are attributed to surface responses. Initially, the surface roughness and brittleness of the coating layer are worn away. Despite efforts to eliminate certain high-hardness waste materials from the environment using centrifugal force, some residues persist between the ball and the substrate. The abrasive effect of wear debris, combined with the high surface hardness of the coating, causes a sudden increase in the coefficient of friction after 6000 cycles. As the surface roughness and brittleness diminish, leading to decreased debris from the coating layer, the friction coefficient stabilizes during long cycling periods (6000–19,000 cycles). A slight decline in the friction coefficient until 20,000 cycles occurs when debris accumulates, with some adhering to the surface. Ultimately, with the removal of some debris by centrifugal force, the friction coefficient returns to an average value until the end of the experiment.

**Figure 14:**
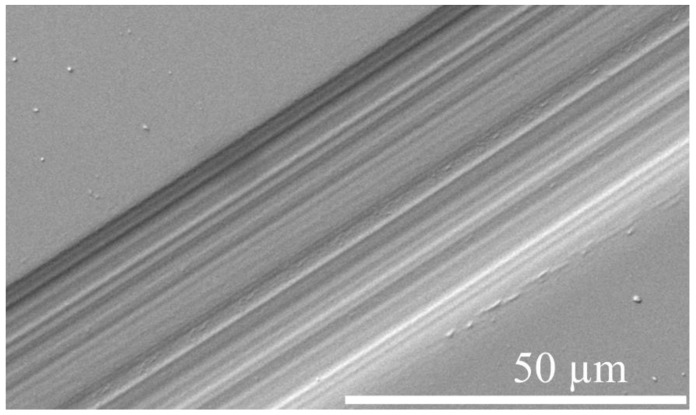
SEM image of the wear track of TiZrMoC S3 after wear tests.

Figure 14 displays the wear tracks on the surface of coating S3, examined using SEM with secondary electron imaging. The wear trace width after 30,000 cycles is approximately 45.5 μm. A detailed examination of the track reveals that damage is most prominent in the central regions, gradually diminishing toward the border zones. The majority of the track exhibits abrasive wear scratches that align with the direction of the ball path, indicating that the primary wear mechanism for the coating is abrasive wear. This observation suggests that lubrication by wear debris, escaping direct contact between the wear-ball and coating surface, could explain the consistent variation in the friction coefficient with changes in wear mechanisms.

The factors influencing wear resistance can be categorized into two groups: extrinsic factors, such as wear ball material, sliding speed, and testing temperature, and intrinsic factors, including coating thickness, roughness, adhesive strength, toughness, and hardness. In this study, the pin-on-disk test maintained fixed extrinsic factors, allowing for a focus solely on intrinsic factors. Given that the coating surface roughness is significantly lower than that of the wear-ball, the impact of surface roughness has been disregarded. Hardness plays a critical role in wear resistance, as coatings must be sufficiently hard to withstand the applied load without experiencing penetration by the wear ball during testing, indicating that harder layers may exhibit superior wear resistance. While adhesive strength is another key factor influencing wear resistance, it is not addressed in this study as adhesion tests were not conducted. Consequently, other factors like residual stress and coating thickness may be pivotal in determining wear resistance. Thicker coatings generally require longer deposition times, resulting in greater energy input into the coating, typically leading to densification and decreased wear rates. However, increasing coating thickness can also elevate residual stress levels, potentially compromising adhesion strength and subsequently increasing wear rates. Hence, exclusively associating wear resistance with thickness or residual stress proves challenging.

## 4. Conclusions

TiZrMoC composite coatings were fabricated via dual DC magnetron sputtering under varying graphite target currents, resulting in a single-phase rock-salt structure with pronounced [111] texture. The coatings displayed columnar grains and notable nanoscale features, including nanotwins, stacking faults, and an amorphous carbon layer (0.5–1.5 nm thick). The optimized composition, identified as Ti_60_Zr_25_Mo_15_/C with mixed carbon phases, demonstrated exceptional mechanical and tribological properties. A peak Knoop hardness of 35 GPa was achieved at a graphite target current of 250 mA, while wear tests confirmed stable low-friction coefficients (~0.1) and predominantly abrasive wear mechanisms.

First-principles calculations underscored the structural stability, elastic moduli, and fracture toughness of quaternary carbide alloys. ZrMoC and TiC were identified as forming ideal solid solutions, while TiZrC-MoC and TiMoC-ZrC displayed limited solubility. Enhanced hardness correlated with increased molybdenum and titanium content, particularly in alloys with a valence electron concentration (VEC) > 8.5, whereas fracture toughness improved with molybdenum content.

The comprehensive investigation of TiZrMoC coatings reveals a promising material with exceptional properties. The precise control of composition and microstructure enables the tailoring of hardness, wear resistance, and thermal stability, surpassing conventional titanium alloys. The unique nanostructured features contribute to these outstanding properties, making these coatings suitable for a wide range of applications, including cutting tools, protective coatings, and tribological components. This research paves the way for the development of advanced materials and devices, driving innovation in various industries.

## Figures and Tables

**Table 1:** Deposition parameters for TiZrMoC coatings.

Name	*F_Ar_* ^a^	*P_c_* ^b^	Target 1		Target 2	
	[sccm]	[Pa]	*U* ^c^ [V]	*I* ^d^ [mA]	*U* [V]	*I* [mA]
			Graphite		TiZrMo	
S1	50	0.15	460	150	300	200
S2	50	0.15	480	200	300	200
S3	50	0.15	510	250	310	200
S4	50	0.15	540	300	320	200

^a^ flow rate of argon; ^b^ working pressure in the chamber; ^c^ voltage on the magnetron; ^d^ magnetron current.

**Table 4:** Lattice parameter (a), cell volume deviation relative to the cell volume of TiC (Δ*V* = [*V*_Alloys_ − *V*_TiC_]/*V*_TiC_), mixing energy (*E_MIX_*), Hill bulk (*B*), shear (*G*), Young (*E*) moduli, Vickers hardness (*H_V_*), Poisson ratio (*ν*), *B*/*G* ratio, and fracture toughness (*K_IC_*) of carbides in the TiZrMoC system. Data shown are from the computational study.

Phase	*a*, [Å]	Δ*V*, [%]	*E_MIX_*, [eV/at]	*B*, [GPa]	*G*, [GPa]	*E*, [GPa]	*B*/*G*	*ν*	*H_V_*, [GPa]	*K_IC_*, [MPa·m^1/2^]
TiC	4.317	0	0	245.2	179.1	432.4	1.37	0.21	25.7	3.38
ZrC	4.708	29.7	0	221.7	163.5	393.7	1.36	0.20	24.6	2.99
MoC	2.926 ^a^ 2.840	4.7	0	349.0	255.1	615.4	1.37	0.21	-	4.42
Ti_0.5_Zr_0.5_C	4.527	15.3	0.069	233.7	166.1	402.8	1.41	0.21	23.8	3.11
Ti_0.5_Mo_0.5_C	4.343	1.8	0.068	295.9	190.6	470.7	1.55	0.23	22.8	4.12
Zr_0.5_Mo_0.5_C	4.543	16.5	0.116	238.8	130.6	331.4	1.83	0.27	14.0	2.85
Ti_0.5_Zr_0.25_Mo_0.25_C	4.430	8.1	0.053	258.2	180.4	439.0	1.43	0.22	24.6	3.54
Ti_0.25_Zr_0.5_Mo_0.25_C	4.528	15.4	0.070	246.3	167.7	410.0	1.47	0.22	22.5	3.29
Ti_0.25_Zr_0.25_Mo_0.5_C	4.448	9.4	0.103	258.3	167.8	412.3	1.54	0.23	21.2	3.42
Ti_0.33_Zr_0.33_Mo_0.33_C	4.467	10.8	0.078	259.6	173.2	425.1	1.50	0.23	22.4	3.50

^a^ *a* and *c* values, respectively. The tolerance of calculated elastic moduli did not exceed ±2.5%. The value of HV for the hexagonal MoC phase is lacking in this table since this phase is unstable at shear stress, much less than the estimated hardness of 37.6 GPa [51].

**Table 5:** Test parameters for the friction coefficient measurement of the TiZrMoC S3 sample.

Area [µm^2^]	Radius [mm]	Total Cycles	Load [N]	Distance [m]	Volume Lost [mm^3^]	Wear Factor
168.64	4.5	30,000	0.75	838.23	0.0047	2.119 × 10^−7^

## Data Availability

The data that support the findings of this study are available from the corresponding author upon request.

## Supplementary Materials

The following supporting information can be downloaded at https://www.mdpi.com/article/10.3390/nano14241986/s1: Figure S1: SIMS depth element profiles of TiZrMoC film (sample S3) corresponding to the start of the growth of a layer with 50% of TiC, 25% of ZrC and 25% of MoC composition (in logarithmic scale); Figure S2: SIMS depth element profiles of TiZrMoC S3 corresponding to the growth of the layer with 50% of TiC, 25% of ZrC and 25% of MoC final composition (in linear scale); Figure S3: DF TEM image of TiZrMoC S3 along the (220) reflection; Figure S4: EDX mapping of the wear track of TiZrMoC S3 after the wear test with WC counter-sample.
