# Supplementary material for: Tunable TiZrMoC Coatings: A Comprehensive Study of Microstructure, Mechanical Properties, and Wear Resistance
Source: Nanomaterials (Basel). 2024 Dec 11;14(24):1986. doi: 10.3390/nano14241986 (PMC11728635; doi:10.3390/nano14241986)
Supplement: Supplementary file 1 [file nanomaterials-14-01986-s001.zip › nanomaterials-3335234-supplementary.pdf]

Supporting Information

Tunable TiZrMoC Coatings: A Comprehensive Study of Microstructure, Mechanical Properties, and Wear Resistance

Alexander Pogrebnjak [1,2,*], Volodymyr Buranych [2], Volodymyr Ivashchenko [3], Svitlana Borba-Pogrebnjak [1], Olga Maksakova [2], Maria Caplovicová [4], Alexander Goncharov [1,2], Alexei Onoprienko [3], Petro Skrynskyy [3], Martin Sahul [2], Piotr Konarski [5], Piotr Budzynski [6], Mariusz Kaminski [6], Marek Opielak [7], Dominik Flock [8], Vasiliy Pelenovich [9,10] and Yang Bing [11]

[1] Biomedical Research Centre, Sumy State University, 116, Kharkivska St., 40007 Sumy, Ukraine
[2] Institute of Materials Science, Faculty of Materials Science and Technology, Slovak University of Technology, J. Bottu 25, 917 24 Trnava, Slovakia
[3] Frantsevich Institute for Problems of Materials Sciences, NAS of Ukraine, Krzhyzhanovsky 3, 03142 Kyiv, Ukraine
[4] Centre for Nanodiagnostics of Materials, Slovak University of Technology in Bratislava, Vazovova 5, 812 43 Bratislava, Slovakia
[5] Łukasiewicz Research Network–Tele and Radio Research Institute, 11, Ratuszowa St., 03-450 Warsaw, Poland
[6] Faculty of Mechanical Engineering, Lublin University of Technology, Nadbystrzycka Str. 36, 20-618 Lublin, Poland
[7] Faculty of Transportation and Information Technology, WSEI University, 4, Projektowa Str., 20-209 Lublin, Poland
[8] Institute of Materials Science and Engineering, Ilmenau University of Technology, Gustav-Kirchhoff Str. 1, 98693 Ilmenau, Germany
[9] Hubei Key Laboratory of Electronic Manufacturing and Packaging Integration, Wuhan University, Wuhan 430072, China
[10] Institute of Technological Sciences, Wuhan University, Wuhan 430072, China
[11] School of Power & Mechanical Engineering, Wuhan University, Wuhan 430072, China
* Correspondence: oleksandr.pohrebniak@stuba.sk

1. Introduction

Ion current ratio values are obtained by dividing the product of the raw secondary ion current “I_x” and the corresponding coefficient “k” by the sum of such products for all tested components.

The obtained formula is as follows:

Ion ratio = $(I_x \times k / \Sigma I_x \times k) \times 100\%$

The values of the “k” coefficients were selected arbitrarily.

Figure S1 shows the SIMS depth element profiles of TiZrMoC film (sample S3) corresponding to the growth of the layer with 50% of TiC, 25% of ZrC and 25% of MoC in the final composition. Here, “k” factors were selected such that the uniform composition of the carbide layer (obtained after a certain time of deposition) was equal to the proportion of 50% TiC, 25% ZrC and 25% MoC.

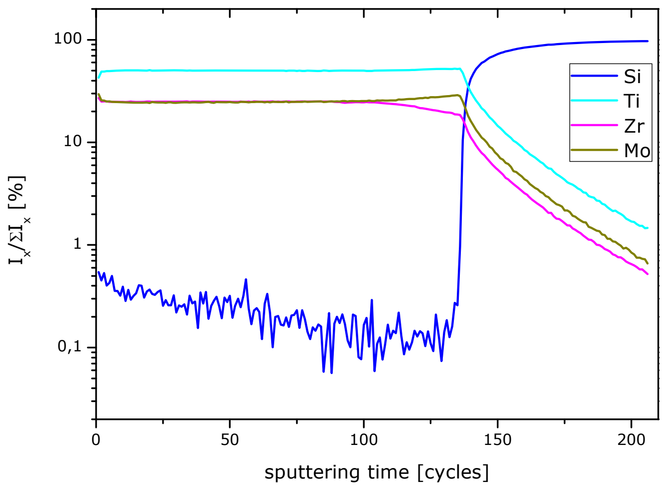

Figure S1. SIMS depth element profiles of TiZrMoC film (sample S3) corresponding to the growth of the layer with 50% of TiC, 25% of ZrC and 25% of MoC final composition (in logarithmic scale).

The secondary ion mass spectrometry (SIMS) analysis indicated variations in intensity between the metal elements and their compounds (Ti, Zr, and Mo). Zr and Mo displayed consistent signal intensities across the metal and its carbide compositions. Furthermore, the M^+ signals exhibited higher intensities attributed to the matrix effect compared to the metal-carbide (MC^+) or metal-oxide (MO^+) signals. The SIMS depth profile examination involved positive ions with the respective analyzed masses of ^{12}C, ^{14}N, ^{16}O, ^{30}Si, ^{49}Ti, ^{90}Zr, and ^{96}Mo. **Figure 2,** shown in the main text, presents the acquired SIMS depth profiles depicting the elemental distribution within the TiZrMoC coating.

The obtained results show that the deposited TiZrMoC film has a surface gradient layer composed of three carbides. The thickness of this gradient layer is about a quarter of the total thickness of the entire film deposited on silicon substrate.

Figure S2 shows SIMS profiles of TiZrMoC film (sample S3) when the carbide layer deposition process started with the composition of 50% TiC, 25% ZrC and 25% MoC. We see the stable concentration of the compositional elements Ti, Zr and Mo. The concentration intensity of Ti is approximately 45 %, when Zr is 32% and Mo is 22 %. A small increase of 1.5 – 2% in Ti and Mo concentration is seen when the sputtering depth reached the close to the substrate region, while the concentration of Zr decreases at approximately 5 %.

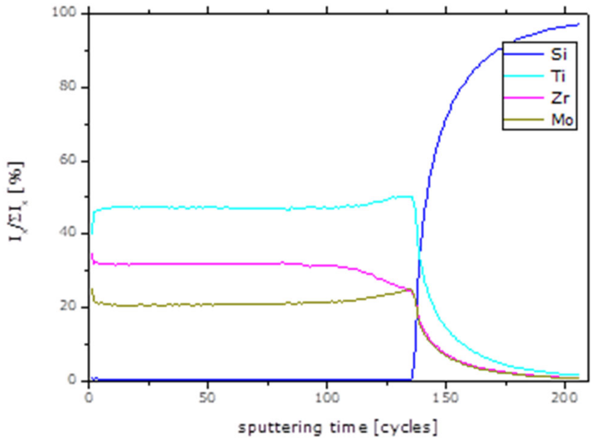

Figure S2. SIMS depth element profiles of TiZrMoC film (sample S3) corresponding to the start of the growth of a layer with 50% of TiC, 25% of ZrC and 25% of MoC composition (in linear scale).

Figure S3 shows DF TEM image of the sample S3 recorded using (220) reflection. A strong crystallographic texture in [111] directions and columnar crystals with diameter from 20 nm to 40 nm are manifested.

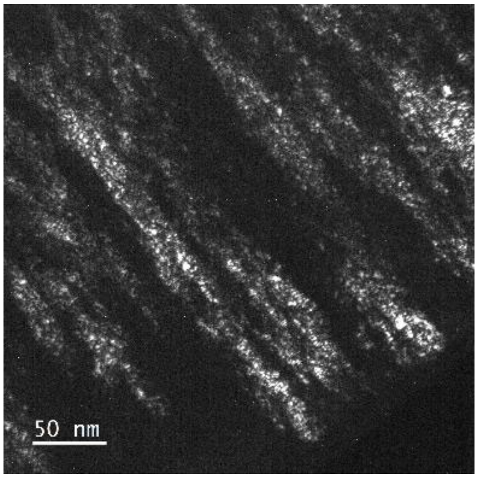

Figure S3. DF TEM image of TiZrMoC S3 along the (220) reflection.

Figure S4 shows the EDX maps obtained for the sample S3 after the wear test for Ti, Zr, Mo, C, Si and O elements. It is obvious that compositional elements are distributed uniformly through the wear track. The concentration contrasts of Zr and Si elements are higher at the middle of the wear track, while for Mo and C, it is higher at the edges. The oxide distribution is non-uniform. One can see that it is higher concentrated in the middle of the wear track in the wave-like form, and its concentration decreases in the direction to the edges of the wear track.

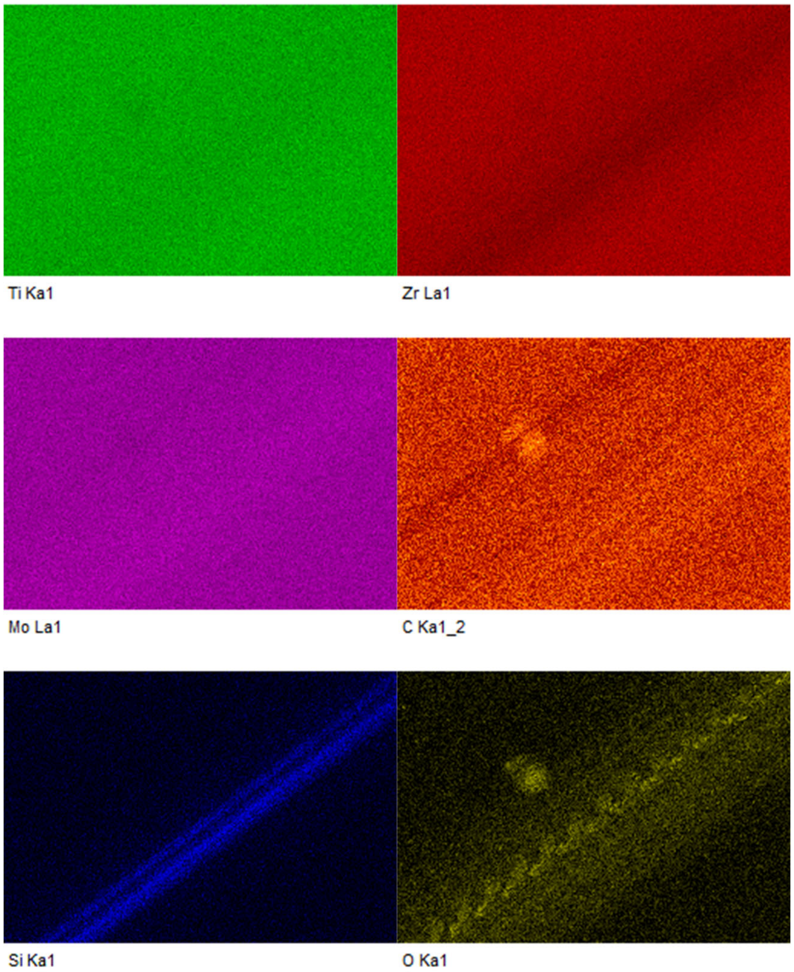

Figure S4. EDX mapping of the wear track of TiZrMoC S3 after the wear test with WC counter-sample.
